# Editorial for the Special Issue “Advances in Cattle, Sheep, and Goats Molecular Genetics and Breeding”

**DOI:** 10.3390/genes16070826

**Published:** 2025-07-16

**Authors:** Xiukai Cao

**Affiliations:** Joint International Research Laboratory of Agriculture and Agri-Product Safety of Ministry of Education of China, Yangzhou University, Yangzhou 225009, China; cxkai0909@163.com

The landscape of livestock breeding has dramatically shifted with the rise of molecular genetics, offering unprecedented insights into the genomic underpinnings of complex traits in domesticated animals. Among these, cattle, sheep, and goats—our three primary ruminant species—are now at the forefront of intensive genomic research. Their economic significance in dairy, meat, and fiber production, alongside their critical role in global food security and rural livelihoods, makes this focus particularly vital. This Special Issue, entitled “Advances in Cattle, Sheep, and Goats Molecular Genetics and Breeding”, amasses 14 original contributions that collectively illuminate the path forward for genetic improvement through multi-omics approaches, high-resolution genotyping, transcriptome analyses, and sophisticated bioinformatics techniques. This collection offers a comprehensive review of how modern tools in molecular and computational biology are being harnessed to unravel traits such as disease resistance, reproductive efficiency, growth, and environmental adaptation. A clear trend emerging from these studies is a rapid transition in livestock genetic improvement: from selection based purely on observable traits to decisions informed by an animal’s genetic makeup.

Several articles focus on growth-related traits, a key target in small ruminant production. Li et al. conducted a genome-wide association study (GWAS) in Hu sheep, identifying 13 candidate genes—including *FGF9*, *BMPR1B*, and *JAK2*—associated with birth, weaning, and monthly weights from 3 to 7 months of age [1]. Their results provide molecular markers for enhancing growth performance in sheep breeding programs. Similarly, Yang et al. studied Hulunbuir sheep and identified genetic regions significantly associated with body size traits such as chest girth and hip width. Notably, genes such as *SLC9C1*, *VSTM2A*, and *FRG1* were associated with morphogenesis [2]. These findings offer valuable insights for phenotype-based selection in native sheep breeds. Cao et al. addressed structural variants in sheep by investigating copy number variations (CNVs) in the *NSMF* gene across three Chinese breeds [3]. Their study found strong associations between *NSMF* CNVs and body weight and chest width. This demonstrates the importance of exploring structural genomic diversity alongside SNP variation to improve the accuracy of selection for growth traits.

Reproductive traits remain a priority in breeding programs for sheep and goats. Zhang et al. explored associations between *BMP15* and *GDF9* polymorphisms and litter size in Hu sheep [4]. Their study confirmed that variants in these oocyte-derived growth factors significantly affect prolificacy, consistent with earlier findings in other small ruminants. These genes are promising targets for improving reproductive efficiency through molecular selection.

The conservation of genetic diversity in local cattle and goat populations is another important theme. Hervás-Rivero et al. performed a runs of homozygosity (ROH) analysis across ten Spanish cattle breeds and identified shared ROH islands containing genes involved in milk production, immunity, and stress response [5]. These findings provide insights into historical selection pressures and genetic drift in autochthonous cattle populations. Wirth et al. used ROH analysis to detect selection in German Brown cattle and highlighted genes linked to milk composition, disease resistance, and reproductive performance [6]. Their data confirm breed-specific selection pressures and support the refinement of breeding schemes in dual-purpose cattle.

Deniskova et al. focused on the endangered Orenburg goat breed, using SNP genotyping to evaluate genetic diversity and structure [7]. The study revealed moderate genetic differentiation and emphasized the need for urgent conservation efforts. Their work offers a framework for recovery plans and germplasm preservation strategies. In parallel, HuangFu et al. used whole-genome sequencing to characterize diversity and selection signatures in Matou goats [8]. They identified regions under positive selection enriched in genes related to reproduction and environmental adaptation, providing genomic evidence for the unique value of this indigenous breed.

The health and disease dimension was explored by Wang et al., who conducted transcriptomic profiling of mammary tissue in Xinjiang Brown cattle to identify resistance mechanisms against mastitis, a costly disease in dairy production [9]. The authors found that genes involved in inflammatory and immune signaling—such as *CD14*, *TLR4*, and *CCL5*—were differentially expressed between resistant and susceptible animals. Their study provides a valuable genomic resource for developing disease-resistant dairy breeds. In the context of parasitic resistance, Vera et al. studied gastrointestinal parasite burden in Australian Merino sheep [10]. By analyzing phenotypic resistance data alongside genotypic information, the authors mapped genomic regions related to host resistance. Key candidate genes included those involved in innate immunity and epithelial barrier integrity. This study adds to the growing body of work aimed at reducing anthelmintic reliance through genetic selection.

Functional genomics and gene regulation are also featured prominently in this collection. Chen et al. reviewed the role of long noncoding RNAs (lncRNAs) in adipogenic differentiation and highlighted their regulatory interactions with PPARG and C/EBPα [11]. The study provided a mechanistic overview of how lncRNAs affect fat deposition, which has direct implications for meat quality improvement. In another functional study, Wang et al. investigated *CRABP1* in dermal papilla cells of Hu sheep and demonstrated that it enhances cell proliferation through the Wnt/β-catenin pathway [12]. Their findings support a role for *CRABP1* in wool follicle development, offering new molecular insights into fiber trait improvement. Wang et al. examined the *PPARG* gene in buffalo and characterized its splicing variants in mammary tissue [13]. Their findings suggest that different isoforms of *PPARG* have distinct expression profiles during lactation and play functional roles in milk fat synthesis, opening new avenues for lactation biology in non-model ruminants.

Behavioral traits, such as temperament, are gaining traction in livestock breeding due to their influence on animal welfare, management, and productivity. Ruiz-De-La-Cruz et al. conducted a systematic review and interaction network analysis to identify the genes and biological pathways associated with bovine temperament [14]. Their findings implicated neurotransmission and neuroendocrine genes such as *AVPR1A*, *DRD4*, and *SLC6A4*, providing potential genomic markers for behavioral selection.

Collectively, these 14 articles exemplify the power of integrating molecular tools into ruminant breeding and genetic conservation. Whether through GWAS, transcriptomics, ROH mapping, or functional gene analysis, the studies presented here provide both fundamental insights and practical applications. Key themes include the molecular dissection of growth and reproduction, structural variation analysis, disease and parasite resistance, and the genetic basis of temperament and adaptation. This Special Issue contributes not only to the understanding of trait architecture in cattle, sheep, and goats but also to the development of sustainable, genomics-informed breeding programs. The diversity of approaches and species represented underscores the continued relevance of molecular genetics in improving livestock performance, health, and resilience under evolving production systems.

We thank all authors and reviewers who contributed to this Special Issue and hope that this collection will serve as a valuable reference for researchers and breeders working across disciplines and species.
